# 1*H*-Imidazol-3-ium-4-carboxyl­ate

**DOI:** 10.1107/S1600536811052998

**Published:** 2011-12-17

**Authors:** Qiang Cao, Bao-Rong Duan, Bin Zhu, Zhen Cao

**Affiliations:** a College of Chemistry and Life Science, Weinan Normal University, 714000 Weinan, Shaanxi, People’s Republic of China; b Chemistry and Chemical Engineering College, Yantai University, 264005 Yantai, Shandong, People’s Republic of China; c Engineering Company Limited of China Railway and Airport Group, 714000 Weinan, Shaanxi, People’s Republic of China; d Shaanxi Railway Institute, 714000 Weinan, Shaanxi, People’s Republic of China

## Abstract

In the title compound, C_4_H_4_N_2_O_2_, both imidazole N atoms are protonated and carboxyl­ate group is deprotonated, resulting in a zwitterion. The mol­ecule is essentially planar, with an r.m.s. deviation of 0.012 (1) Å. In the crystal, N—H⋯O hydrogen bonds and π–π stacking inter­actions [centroid–centroid distance = 3.674 (2) Å] between the imidazole rings link the mol­ecules into a three-dimensional supra­molecular network.

## Related literature

For general background to the construction of coordination polymers based on 1*H*-imidazole-4,5-dicarb­oxy­lic acid, see: Alkordi, Liu *et al.* (2008 ▶); Alkordi, Brant *et al.* (2009 ▶); Gu *et al.* (2010 ▶); Lu *et al.* (2006 ▶); Nouar *et al.* (2009 ▶); Wang *et al.* (2010 ▶). For related complexes with 1*H*-imidazole-4-carb­oxy­lic acid, see: Haggag (2005 ▶); Starosta & Leciejewicz (2006 ▶); Gryz *et al.* (2007 ▶); Yin *et al.* (2009 ▶); Shuai *et al.* (2011 ▶); Zheng *et al.* (2011 ▶). For the synthesis of 1*H*-imidazole-4-carb­oxy­lic acid, see: Davis *et al.* (1982 ▶).
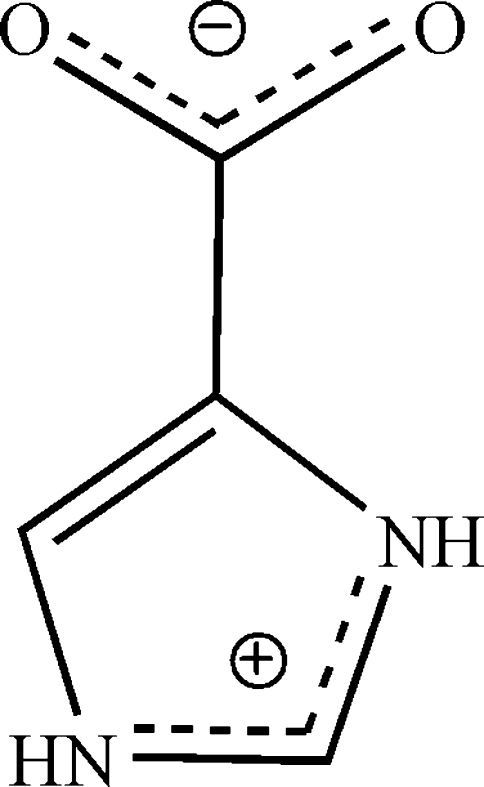


## Experimental

### 

#### Crystal data

C_4_H_4_N_2_O_2_
*M* *_r_* = 112.09
Orthorhombic, 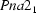

*a* = 10.474 (6) Å
*b* = 11.676 (7) Å
*c* = 3.674 (2) Å
*V* = 449.3 (5) Å^3^
*Z* = 4
Mo *K*α radiation
μ = 0.14 mm^−1^
*T* = 298 K
0.25 × 0.21 × 0.18 mm

#### Data collection

Bruker APEXII CCD area-detector diffractometer
Absorption correction: multi-scan (*SADABS*; Sheldrick, 1996 ▶) *T*_min_ = 0.967, *T*_max_ = 0.976
2280 measured reflections
510 independent reflections
480 reflections with *I* > 2σ(*I*)
*R*_int_ = 0.023

#### Refinement

*R*[*F* ^2^ > 2σ(*F* ^2^)] = 0.029
*wR*(*F* ^2^) = 0.083
*S* = 1.10
510 reflections
73 parameters
1 restraint
H-atom parameters constrained
Δρ_max_ = 0.12 e Å^−3^
Δρ_min_ = −0.19 e Å^−3^

### 

Data collection: *APEX2* (Bruker, 2008 ▶); cell refinement: *SAINT* (Bruker, 2008 ▶); data reduction: *SAINT*; program(s) used to solve structure: *SHELXS97* (Sheldrick, 2008 ▶); program(s) used to refine structure: *SHELXL97* (Sheldrick, 2008 ▶); molecular graphics: *SHELXTL* (Sheldrick, 2008 ▶); software used to prepare material for publication: *SHELXTL*.

## Supplementary Material

Crystal structure: contains datablock(s) I, global. DOI: 10.1107/S1600536811052998/ld2040sup1.cif

Structure factors: contains datablock(s) I. DOI: 10.1107/S1600536811052998/ld2040Isup2.hkl

Supplementary material file. DOI: 10.1107/S1600536811052998/ld2040Isup3.cml

Additional supplementary materials:  crystallographic information; 3D view; checkCIF report

## Figures and Tables

**Table 1:** Hydrogen-bond geometry (Å, °)

*D*—H⋯*A*	*D*—H	H⋯*A*	*D*⋯*A*	*D*—H⋯*A*
N2—H2⋯O2^i^	0.86	1.82	2.648 (2)	160
N1—H1⋯O1^ii^	0.86	1.91	2.736 (2)	161

Symmetry codes: (i) 
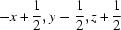
; (ii) 
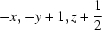
.

**Fig. 1.**
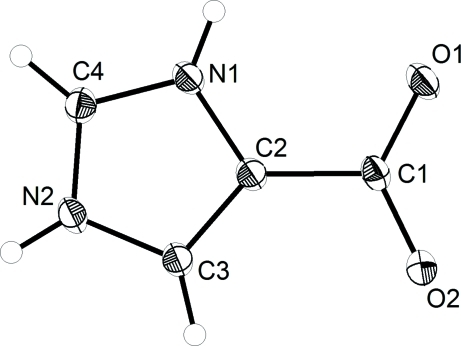
The structure of the title compound, showing the atom-labelling scheme and displacement ellipsoids drawn at the 30% probability level.

**Fig. 2.**
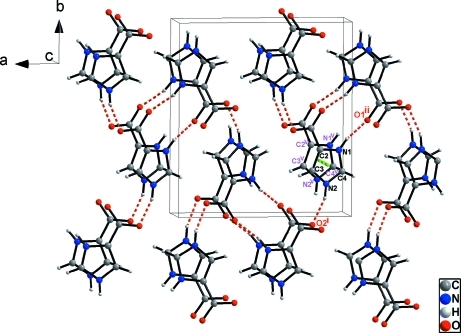
A view showing part of the three-dimensional supramolecular network linked by N–H···O hydrogen bonds and π-π stacking interactions. Hydrogen bonds and π-π stacking interactions are shown as dashed lines. Symmetry codes: (i) -x + 1/2, y - 1/2, z + 1/2; (ii) -x, -y + 1, z + 1/2; (v) x, y, z + 1.

## supplementary crystallographic information

### Comment

The organic ligands containing N and O donors, especially the *N*-heterocyclic carboxylates, are ideal candidates for constructing novel metal coordination polymers, because of their versatile coordination modes and potential hydrogen bonding donors and acceptors. Particular attention has been paid to the 1*H*-imidazole-4,5-dicarboxylic acid ligand (H_3_IDC), because it can coordinate with metal ions in different coordination fashions to offer a series of complexes with diverse structures and interesting properties (Alkordi, Liu *et al.*, 2008; Alkordi, Brant *et al.*, 2009; Gu *et al.*, 2010; Lu *et al.*, 2006; Nouar *et al.*, 2009; Wang *et al.*, 2010). Recently, an analogue of H_3_IDC, 1*H*-imidazole-4-carboxylic acid (H_2_IMC), has also been used to prepare new coordination polymers (Haggag, 2005; Starosta & Leciejewicz, 2006; Gryz *et al.*, 2007; Yin *et al.*, 2009; Shuai *et al.*, 2011; Zheng *et al.*, 2011). However, the crystal structure of H_2_IMC ligand has not been determined. With this in mind, we attempteted to obtain its crystal structure that is reported here.

As illustrated in Fig. 1, the title compound, C_4_H_4_N_2_O_2_, crystallizes as a zwitterion in which the imidazole N atom is protonated and the carboxylate group is deprotonateded. In the crystal structure, intermolecular N2—H2···O2^i^ and N1—H1···O1^ii^ hydrogen bonds (Table 1) [symmetry code: (i) -*x* + 1/2, *y* - 1/2, *z* + 1/2; (ii) -*x*,-*y* + 1, *z* + 1/2] between the imidazole N—H groups and carboxylate O atoms link the molecules into a three-dimensional supramolecular network (Fig. 2). Moreover, the crystal structure is further stabilized by π-π stacking interactions between neighbouring imidazole rings [N2—C4—N1—C2—C3 and N2^v^—C4^v^—N1^v^—C2^v^—C3^v^, symmetry code: (v) *x*, *y*, *z* + 1], with centroid···centroid distances of 3.674 (2) Å (Fig. 2).

### Experimental

The compound was synthesized from 1*H*-imidazole-4,5-dicarboxylic acid according to the method reported in the literature (Davis *et al.*, 1982). Colourless single crystals suitable for X-ray diffraction were prepared by slow evaporation of a solution of the title compound in water at room temperature.

### Refinement

All non-hydrogen atoms were assigned anisotropic displacement parameters in the refinement. The zwitter-ionic structure was established from a difference Fourier synthesis. Consequently, all hydrogen atoms were positioned geometrically and refined using a riding model, with C—H = 0.93 Å and N—H =0.86 Å and with *U*_iso_(H) =1.2 *U*_eq_(C, N). Since this is a light atom structure (does not contain any atoms heavier than Si) and since the data collection was carried out using Mo radiation, it was not possible to unambiguously determine the absolute configuration of this molecule. In the absence of significant anomalous scattering effects Friedel pairs have been merged.

### Crystal data

**Table:**

C_4_H_4_N_2_O_2_	*F*(000) = 232
*M**_r_* = 112.09	*D*_x_ = 1.657 Mg m^−^^3^
Orthorhombic, *P**n**a*2_1_	Mo *K*α radiation, λ = 0.71073 Å
Hall symbol: P 2c -2n	Cell parameters from 1380 reflections
*a* = 10.474 (6) Å	θ = 2.6–27.0°
*b* = 11.676 (7) Å	µ = 0.14 mm^−^^1^
*c* = 3.674 (2) Å	*T* = 298 K
*V* = 449.3 (5) Å^3^	Block, colourless
*Z* = 4	0.25 × 0.21 × 0.18 mm

### Data collection

**Table:**

Bruker APEXII CCD area-detector diffractometer	510 independent reflections
Radiation source: fine-focus sealed tube	480 reflections with *I* > 2σ(*I*)
graphite	*R*_int_ = 0.023
phi and ω scans	θ_max_ = 26.0°, θ_min_ = 2.6°
Absorption correction: multi-scan (*SADABS*; Sheldrick, 1996)	*h* = −12→12
*T*_min_ = 0.967, *T*_max_ = 0.976	*k* = −14→12
2280 measured reflections	*l* = −4→4

### Refinement

**Table:**

Refinement on *F*^2^	Primary atom site location: structure-invariant direct methods
Least-squares matrix: full	Secondary atom site location: difference Fourier map
*R*[*F*^2^ > 2σ(*F*^2^)] = 0.029	Hydrogen site location: inferred from neighbouring sites
*wR*(*F*^2^) = 0.083	H-atom parameters constrained
*S* = 1.10	*w* = 1/[σ^2^(*F*_o_^2^) + (0.0578*P*)^2^ + 0.0406*P*] where *P* = (*F*_o_^2^ + 2*F*_c_^2^)/3
510 reflections	(Δ/σ)_max_ < 0.001
73 parameters	Δρ_max_ = 0.12 e Å^−^^3^
1 restraint	Δρ_min_ = −0.19 e Å^−^^3^

### Special details

**Table:**

Geometry. All e.s.d.'s (except the e.s.d. in the dihedral angle between two l.s. planes) are estimated using the full covariance matrix. The cell e.s.d.'s are taken into account individually in the estimation of e.s.d.'s in distances, angles and torsion angles; correlations between e.s.d.'s in cell parameters are only used when they are defined by crystal symmetry. An approximate (isotropic) treatment of cell e.s.d.'s is used for estimating e.s.d.'s involving l.s. planes.
Refinement. Refinement of *F*^2^ against ALL reflections. The weighted *R*-factor *wR* and goodness of fit *S* are based on *F*^2^, conventional *R*-factors *R* are based on *F*, with *F* set to zero for negative *F*^2^. The threshold expression of *F*^2^ > σ(*F*^2^) is used only for calculating *R*-factors(gt) *etc*. and is not relevant to the choice of reflections for refinement. *R*-factors based on *F*^2^ are statistically about twice as large as those based on *F*, and *R*- factors based on ALL data will be even larger.

### Fractional atomic coordinates and isotropic or equivalent isotropic displacement parameters (Å^2^)

**Table:**

	*x*	*y*	*z*	*U*_iso_*/*U*_eq_	
C2	0.15183 (19)	0.33109 (16)	0.6250 (7)	0.0265 (5)	
C1	0.20900 (19)	0.44287 (17)	0.5153 (6)	0.0268 (5)	
N2	0.10851 (15)	0.15065 (14)	0.7501 (6)	0.0302 (5)	
H2	0.1149	0.0774	0.7660	0.036*	
C4	0.0113 (2)	0.21278 (18)	0.8693 (7)	0.0301 (5)	
H4	−0.0610	0.1844	0.9853	0.036*	
O2	0.31910 (13)	0.43682 (12)	0.3734 (5)	0.0361 (5)	
O1	0.14526 (14)	0.53104 (13)	0.5736 (6)	0.0367 (5)	
N1	0.03417 (16)	0.32279 (14)	0.7956 (6)	0.0284 (5)	
H1	−0.0158	0.3791	0.8456	0.034*	
C3	0.1970 (2)	0.22218 (17)	0.5977 (7)	0.0280 (5)	
H3	0.2743	0.2003	0.4940	0.034*	

### Atomic displacement parameters (Å^2^)

**Table:**

	*U*^11^	*U*^22^	*U*^33^	*U*^12^	*U*^13^	*U*^23^
C2	0.0254 (9)	0.0223 (10)	0.0320 (13)	0.0003 (8)	−0.0016 (10)	0.0003 (10)
C1	0.0295 (11)	0.0190 (9)	0.0319 (13)	0.0011 (8)	−0.0047 (9)	0.0035 (9)
N2	0.0323 (9)	0.0181 (8)	0.0401 (11)	−0.0007 (7)	−0.0033 (9)	0.0011 (9)
C4	0.0275 (10)	0.0271 (10)	0.0358 (14)	−0.0035 (8)	−0.0004 (9)	0.0032 (11)
O2	0.0324 (8)	0.0235 (7)	0.0524 (12)	−0.0013 (6)	0.0078 (8)	0.0043 (9)
O1	0.0352 (8)	0.0203 (7)	0.0545 (12)	0.0047 (6)	−0.0018 (8)	0.0025 (9)
N1	0.0279 (9)	0.0203 (8)	0.0371 (11)	0.0024 (7)	−0.0016 (9)	−0.0004 (8)
C3	0.0261 (9)	0.0222 (10)	0.0357 (14)	−0.0005 (8)	−0.0022 (10)	0.0017 (10)

### Geometric parameters (Å, °)

**Table:**

C2—C3	1.361 (3)	N2—C3	1.368 (3)
C2—N1	1.386 (3)	N2—H2	0.8600
C2—C1	1.491 (3)	C4—N1	1.334 (3)
C1—O1	1.246 (3)	C4—H4	0.9300
C1—O2	1.267 (3)	N1—H1	0.8600
N2—C4	1.324 (3)	C3—H3	0.9300
			
C3—C2—N1	106.10 (17)	N2—C4—N1	108.81 (19)
C3—C2—C1	131.2 (2)	N2—C4—H4	125.6
N1—C2—C1	122.71 (17)	N1—C4—H4	125.6
O1—C1—O2	127.19 (19)	C4—N1—C2	108.57 (17)
O1—C1—C2	117.49 (18)	C4—N1—H1	125.7
O2—C1—C2	115.32 (18)	C2—N1—H1	125.7
C4—N2—C3	108.78 (17)	C2—C3—N2	107.74 (19)
C4—N2—H2	125.6	C2—C3—H3	126.1
C3—N2—H2	125.6	N2—C3—H3	126.1
			
C3—C2—C1—O1	−180.0 (3)	C3—C2—N1—C4	0.5 (3)
N1—C2—C1—O1	−1.9 (3)	C1—C2—N1—C4	−178.0 (2)
C3—C2—C1—O2	−0.5 (4)	N1—C2—C3—N2	−0.2 (3)
N1—C2—C1—O2	177.6 (2)	C1—C2—C3—N2	178.1 (2)
C3—N2—C4—N1	0.4 (3)	C4—N2—C3—C2	−0.1 (3)
N2—C4—N1—C2	−0.6 (3)		

### Hydrogen-bond geometry (Å, °)

**Table:**

*D*—H···*A*	*D*—H	H···*A*	*D*···*A*	*D*—H···*A*
N2—H2···O2^i^	0.86	1.82	2.648 (2)	160.
N1—H1···O1^ii^	0.86	1.91	2.736 (2)	161.

Symmetry codes: (i) −*x*+1/2, *y*−1/2, *z*+1/2; (ii) −*x*, −*y*+1, *z*+1/2.
